# The Association between Previous Antibiotic Consumption and SARS-CoV-2 Infection: A Population-Based Case-Control Study

**DOI:** 10.3390/antibiotics12030587

**Published:** 2023-03-15

**Authors:** Matan Dugot, Eugene Merzon, Shai Ashkenazi, Shlomo Vinker, Ilan Green, Avivit Golan-Cohen, Ariel Israel

**Affiliations:** 1 Adelson School of Medicine, Ariel University, Ariel 40700, Israel; emarzon@leumit.co.il; 2 Leumit Health Services, Tel Aviv 64738, Israel; 3 Sackler Faculty of Medicine, Tel Aviv University, Tel Aviv 69978, Israel

**Keywords:** antimicrobial agents, microbiome, respiratory infections, COVID-19, immune modulation

## Abstract

**Background**: The susceptibility to SARS-CoV-2 infection is complex and not yet fully elucidated, being related to many variables; these include human microbiome and immune status, which are both affected for a long period by antibiotic use. We therefore aimed to examine the association of previous antibiotic consumption and SARS-CoV-2 infection in a large-scale population-based study with control of known confounders. **Methods:** A matched case–control study was performed utilizing the electronic medical records of a large Health Maintenance Organization. Cases were subjects with confirmed SARS-CoV-2 infection (*n* = 31,260), matched individually (1:4 ratio) to controls without a positive SARS-CoV-2 test (*n* = 125,039). The possible association between previous antibiotic use and SARS-CoV-2 infection was determined by comparing antibiotic consumption in the previous 6 and 12 months between the cases and controls. For each antibiotic consumed we calculated the odds ratio (OR) for documented SARS-CoV-2 infection, 95% confidence interval (CI), and *p*-value using univariate and multivariate analyses. **Results:** The association between previous antibiotic consumption and SARS-CoV-2 infection was complex and bi-directional. In the multivariate analysis, phenoxymethylpenicillin was associated with increased rate of SARS-CoV-2 infection (OR 1.110, 95% CI: 1.036–1.191) while decreased rates were associated with previous consumption of trimethoprim-sulfonamides (OR 0.783, 95% CI: 0.632–0.971) and azithromycin (OR 0.882, 95% CI: 0.829–0.938). Fluroquinolones were associated with decreased rates (OR 0.923, 95% CI: 0.861–0.989) only in the univariate analysis. Previous consumption of other antibiotics had no significant association with SARS-CoV-2 infection. **Conclusions:** Previous consumption of certain antibiotic agents has an independent significant association with increased or decreased rates of SARS-CoV-2 infection. Plausible mechanisms, that should be further elucidated, are mainly antibiotic effects on the human microbiome and immune modulation.

## 1. Introduction

### 1.1. The Pandemic

The coronavirus disease 2019 (COVID-19), which is caused by the severe acute respiratory syndrome coronavirus 2 (SARS-CoV-2), has spread globally with enormous worldwide impact, and has therefore been declared a pandemic by the World Health Organization (WHO). As of 3 February 2022, the WHO has recorded more than 750 million confirmed cases, including 6.8 million deaths [1]. A recent study concluded that the true number of fatal cases was three times higher [2]. The presentation of SARS-CoV2 infection has an exceptional interindividual variability, ranging from asymptomatic infection in nearly half of the cases to a very severe course with the need of intensive care and leading to long-term complications and deaths [3,4].

### 1.2. Risk Factors for SARS-CoV-2 Infection

SARS-CoV-2 infection has been a major public health problem for about three years with a huge burden on society. It is therefore crucial to elucidate variables that are associated with the infection and its severity, in order to target prevention measures, such as vaccination, social isolation, and special attention to hygiene practices. Demographic factors, including older age, male gender, low socioeconomic status (SES), and ethnicity play a role on the rate and severity of the infections [5,6,7,8], as do certain underlying conditions, including smoking [9], obesity [10], vitamin D deficiency [11], and several underlying diseases [12,13,14,15,16,17].

### 1.3. Human Microbiome and COVID-19

The human microbiome, defined as all the microorganisms that exist inside and on the surface of human body, lives in symbiosis with human systems and plays a key role in host metabolism, physiology, immunology, and even brain function. Imbalance, called dysbiosis, of the human respiratory and intestinal microbiome has been observed in patients with COVID-19 [18]. Examination of the respiratory microbiome of 507 humans, including patients hospitalized with COVID-19, non-COVID patients, and healthy controls, documented that COVID-19 patients had dysbiosis of their upper respiratory microbiome with reduced diversity that correlated with disease severity [19]. Intubated patients had specific lung microbiota with prominent staphylococci [19]. Likewise, several studies found dysbiosis of the intestinal microbiome in patients with COVID-19 with reduction in the Firmicutes phylum and other changes that were correlated with SARS-CoV-2 positivity [20] and disease severity [21,22]. Moreover, the “signature” of the human microbiome has been related to complications, prolonged disease, and mortality from COVID-19 [18,23,24].

### 1.4. Antibiotic Treatment

Antibiotics, a class of medications with diverse mechanisms of action and spectrum of antimicrobial activity, are very commonly used in clinical practice. In addition to their activity on the culprit pathogens, antibiotics have significant and prolonged effects on the human microbiome, mainly by reducing microbial diversity and having a selective pressure which promotes the growth of resistant bacteria [25,26,27]. The reduced diversity and imbalanced composition of the microbiota affects its important functional attributes to host metabolism and physiology, including the gut and systemic immunity [25,28]. Several antibiotic agents have also direct immune modulation activity [29,30,31,32,33,34]. These unintended effects of antibiotic treatment might affect the susceptibility to infectious diseases [27,35,36].

### 1.5. Aim of Study

We therefore aimed to examine the association between previous antibiotic treatment and SARS-CoV-2 infection in a large-scale population-based study, with control of known confounders. Elucidation by the class of antibiotics and the specific antibiotic agents was also performed to shed light on potential pathogenic mechanisms. This pioneering study might lead to new insights on the pathogenic mechanisms involved in SARS-CoV-2 infection.

## 2. Methods

### 2.1. Study Population

This population-based case–control study was conducted at Leumit Health Services (LHS), a large Healthcare Maintenance Organization (HMO) in Israel serving 724,129 persons during the study period from 1 March 2020 to 31 December 2020. LHS has a comprehensive computerized database, which is continuously updated concerning the demographics, medical visits, laboratory tests, hospitalizations, and medication prescriptions of the registered subjects. Prescription records are available from 1998 and include confirmation of purchase by the individual patients. All LHS members have similar general health insurance and equal access to health services. Diagnoses are according to the International Classification of Diseases-9 or 10 (ICD-9 or ICD-10), depending on the date of diagnosis. The validity of the registry has been previously examined and confirmed as high [37].

### 2.2. Definitions

Cases were defined as individuals who had a positive RT-PCR test for SARS-CoV-2 during the period. According to the national criteria for SARS-CoV-2 examination, RT-PCR testing was performed by physician referral after exposure to a patient with confirmed SARS-CoV-2 infection or due to presenting symptoms suggesting COVID-19. Nasopharyngeal swabs were examined for SARS-CoV-2 by a real time RT-PCR assay with internal positive and negative controls, using the COBAS SARS-CoV-2 6800/8800 (Roche Pharmaceuticals, Basel, Switzerland). None of our participants had a SARS-CoV-2 infection before the beginning of the study period, nor had any of them been vaccinated against COVID-19 before or during the study period.

Controls were defined as individuals without a positive SARS-CoV-2 test during the same study period, which were matched to the cases (see below). Levels of SES were defined according to the Israeli Central Bureau of Statistics classification to 20 levels. Previous antibiotic treatment was defined as prescription and purchase of an antibiotic agent by the individual during a defined period before the date of the SARS-CoV-2 RT-PCR test (cases) or the same index date (controls).

### 2.3. Study Design

We conducted a case–control study. For each SARS-CoV-2-positive patient we selected four individuals without a positive SARS-CoV-2 test, matched carefully for age, gender, smoking, family status, ethnic sector, SES, body mass index (BMI), height, overweight category, smoking status, pulse, systolic and diastolic blood pressure, hypertension, and diabetes mellitus. The controls were also matched for selected laboratory results that might affect the rate of SARS-CoV-2 infection, including serum creatinine, estimated glomerular filtration rate [eGFR], and hemoglobin A1C (HbA1C). The strict matching of the control individuals to the cases by multiple relevant demographic, clinical and selected laboratory variables, using advanced tracking methodologies and advanced digital system, minimized the probability of confounding. We did not control for specific less common diseases that might affect susceptibility to SARS-CoV-2 infection, such as asthma, heart diseases, chronic obstructive pulmonary disease (COPD), inflammatory bowel disease (IBD), dementia, underlying malignancy, and attention deficit-hyperactivity disorder (ADHD). These were therefore entered to and examined in the multivariate model to determine the variables that were independently related to SARS-CoV-2 infection. The controls were assigned the same date as the index case.

Exposure to antibiotic medications was obtained from each subject’s electronic record. All antibiotic agents purchased during the study period by the cases and controls were identified according to the ATC codes. To avoid the scenario that the antibiotics were prescribed for symptoms of SARS-CoV-2 infection, antibiotic use was examined until 15 or 30 days before the date of the SARS-CoV-2 PCR testing. We examined the possible association between previous antibiotic treatment and SARS-CoV-2 infection by comparing antibiotic consumption between the cases and control groups.

### 2.4. Statistical Analysis

Statistical analyses were performed using R statistical software version 3.6 (R Foundation for statistical computing). Assumptions were two-sided, with a predefined α of <0.05. Socio-demographic characteristics between the groups of cases and controls were assessed using the Fisher exact test for categorical variables, and the two-tailed Wilcoxon Mann–Whitney U for continuous variables. The primary outcome was the probability of having SARS-CoV-2 infection, examined as related to antibiotic consumption. This was assessed using a logistic regression model to calculate the odds ratios [OR], the corresponding 95% confidence interval [CI], and the significance. Specific diseases that were not matched for and showed different rates between the cases and control groups were entered into and examined with a multivariate analysis, together with previous antibiotic consumption. The Benjamini–Hochberg (BH) procedure was utilized to control the false discovery rate for multiple testing.

The study protocol was approved by the Shamir Medical Center Review Board and the Research Committee of LHS (0129-20-LEU-31.5.2020).

## 3. Results

### 3.1. Study Population

The study population included two groups: 31,260 individuals who had a SARS-CoV-2 infection during the study period which was confirmed by a SARS-CoV-2 positive RT-PCR test and comprised the cases, and a 125,039-individual control group without a positive SARS-CoV-2 positive result during the same study period that were matched 4:1 with the control group.

### 3.2. Demographic Characteristics of the Study Groups

Table 1 shows the demographic characteristics of the groups, documenting a very strict matching with no real demographic differences between the cases and control groups regarding the gender, age, family status, ethnicity, and SES. Details are presented in Table 1.

### 3.3. Clinical Characteristics of the Study Groups

Table 2 reports the basic clinical variables of the groups. As can be seen, the matching was exact with no actual differences between the cases and control groups regarding the smoking status, BMI, categories of overweight, height, pulse, systolic and diastolic blood pressures, hypertension, and diabetes mellitus. The details are presented in Table 2. Specific diseases were not matched for; thus, those significantly affecting the susceptibility to SARS-CoV-2 infections were entered in a multivariate analysis to determine the independence of the association between antibiotic consumption and SARS-CoV-2 infection.

### 3.4. Laboratory Characteristics of the Study Groups

Table 3 shows the basic laboratory results in the cases and control groups. These document the rigorous matching with no evident differences between the groups regarding the levels of serum creatinine, eGFR, and the various serum lipids. Details are presented in Table 3.

### 3.5. Previous Antibiotic Consumption and SARS-CoV-2 Infection

Antibiotics were frequently used in individuals of both groups. The association between antibiotic consumption during several defined periods before the SARS-CoV-2 testing and SARS-CoV-2 infection was examined by comparing antibiotic consumption between the groups of cases and matched controls. Highly significant associations were documented, as some antibiotic agents were associated with decreased rates of SARS-CoV-2 infection, others with increased rates, and others without a significant association, as demonstrated in Table 4A–C. The significant associations were similar among the three periods of previous antibiotic consumption.

Previous consumption of trimethoprim-sulfamethoxazole was associated consistently and significantly with the highest reduction (25–50%) in the rates of SARS-CoV-2 infection. Fluoroquinolones (mainly ciprofloxacin) and azithromycin consumptions were associated with a milder decrease (8–15%) in SARS-CoV-2 infection, which reached consistent statistical significance. The orally administered phenoxymethylpenicillin was the only antibiotic agent whose previous consumption was associated with a significant increased rate of about 10% of SARS-CoV-2 infection.

### 3.6. Multivariate Analysis

To examine whether the significant association found between certain antibiotic agents and SARS-CoV-2 infection were independent, they were examined in a multivariate model, together with underlying diseases that were not matched for and showed different rates between the cases and control groups. As can be seen in Table 5, three antibiotic agents were independently associated with SARS-CoV-2 infection.

## 4. Discussion

### 4.1. New Findings and Their Discussion

In this population-based large-scale study we documented a novel finding that previous consumption of several antibiotic agents was significantly associated with reduced or increased odds for SARS-CoV-2 infection. By establishing a control group strictly matched for demographic and clinical variables and by performing a multivariate analysis with underlying diseases that affect SARS-CoV-2 infection rate, the independent association of certain antibiotic agents with SARS-CoV-2 infection was proven. To the best of our knowledge, this is the first and most comprehensive study to examine this association.

Our findings are in line with previous studies that demonstrated major and prolonged effects of antibiotic treatment on the human microbiome [25,27,35] and that the composition of the microbiome had a considerable impact on the rates of SARS-CoV-2 infection and the severity of COVID-19 [18,19,21,23]. In particular, as the respiratory tract is the entry site of SARS-CoV-2 and the major area of COVID-19, changes in the respiratory microbiome in particular had a major effect on the course of COVID-19 [19,37,38]. Specific “microbial signatures” of the respiratory tract have been linked to COVID-19 [39]. Our findings add an additional specific facet to these observations.

These findings are within the general broader perspective of the relations of the microbiome and the rates of viral infections [40,41,42,43] and their severity [42]. A comprehensive review has shown that dysbiosis of the human microbiome is related to increased rates of viral infections and even suggested advanced manipulations to target gut microbiome [40]; the use of probiotics has also been suggested [43]. The decisive mechanisms involved and the gut–lung axis have been explored using a mouse model. Bifidobacterium pseudolongum NjM1-enriched gut microbiota of mice protected against influenza by acetate production and inflammasome-mediated signaling of interferon production [41]. A detailed study of the oropharyngeal metabolome in pediatric patients with or without influenza A virus pneumonia demonstrated significantly higher levels of sphingolipid and propanoate metabolites between patients with influenza pneumonia and healthy controls [44].

Due to the observational nature of this study, we defined our finding as an association, not necessarily a causality. The matched control group and the logistic regression were designed to control for variables associated with the risk of SARS-CoV-2 infection, but additional unknown variables may play a role. It is also possible, for example, that behavioral differences exist between the cases and control groups, for example, higher/lower adherence to hygiene measures or social distancing during the pandemic. However, the finding that some antibiotic agents were associated with decreased rates of SARS-CoV-2 infection while others with increased rates makes this possibility unlikely, although still conceivable. It seems very unlikely that the infectious disease for which antibiotics were prescribed affected the susceptibility to SARS-CoV-2 infection several weeks or months afterwards. It is feasible that the present findings will stimulate further studies, based mostly on prospective research, to determine the causality.

The susceptibility to SARS-CoV-2 infection is complex and not yet fully elucidated, being likely attributed to a complex interaction between host and environmental risk factors [7,10,12,13,14,15,17,45]. We found that previous consumption of four antibiotic agents—phenoxymethylpenicillin, azithromycin, trimethoprim-sulfamethoxazole (TMP/SMX) and fluoroquinolones—had significant bi-directional associations with SARS-CoV-2 infection rates. It is conceivable that two main general mechanisms, and the interplay between them, are plausible explanations for the influence of previous antibiotic therapy on the susceptibility to SARS-CoV-2 infection and COVID-19.

One major mechanism is probably the effects of antibiotic therapy on the human microbiome, which are significant and long-term and have a secondary impact on the immune response [19,25,27,35]. This is the most likely mechanism by which phenoxymethylpenicillin is associated with a significant and independent increased susceptibility to SARS-CoV-2 infection. This narrow-spectrum penicillin is commonly used and its bactericidal activity encompasses mainly Gram-positive bacteria, aerobic and anaerobic, that are the most common inhabitants of the human microbiome of the upper respiratory tract [19,39]. As the upper respiratory tract microbiome is an important gatekeeper of respiratory infections, phenoxymethylpenicillin-induced dysbiosis and reduced diversity of the local microbiome might increase the susceptibility to SARS-CoV-2 infection. This is in concert with previous observations that dysbiosis of the human respiratory microbiome increased the risk of SARS-CoV-2 infection and severe course of COVID-19 [19,37,38,39], and of other viral infections of the upper respiratory tract such as influenza [38,40].

The other antibiotic agents that were associated with changing rates of SARS-CoV-2 infections were allied with a significant and independent decrease in the infection. A plausible mechanism for such influence is the immune modulation effects exerted by certain antimicrobial agents [28,30,32,34], in addition to the changes in the immune response secondary to the antibiotic-induced altered microbiome.

Azithromycin is well-known for its considerable effects on the immune system [29,30,46]. Its confirmed immunomodulatory effects include reduced production of pro-inflammatory cytokines such as interleukins-8 (IL-8), IL-6, tumor necrotic factor alpha (TNF-α) and matrix metalloproteinases. Azithromycin also modulates macrophage and T-helper functions, causes alterations in autophagy and reduces oxidative stress [29,30,46]. Because of its profound immunomodulatory effects, azithromycin has been proposed for the prevention and treatment of asthma and other inflammatory conditions [29,30].

Furthermore, previous studies also reported antiviral activities of azithromycin, probably by inhibiting the endosome acidification during viral replication and affecting the un-coating step of viral infection [29,47]. Because of these properties, and as inflammation plays a major role in the severity of COVID-19, azithromycin has been suggested as a treatment for this infection, alone or with hydroxychloroquine [29,46], but several studies did not document efficacy of azithromycin in patients with COVID-19 and it is currently not recommended as a treatment for the disease. We have found that previous consumption of azithromycin is associated with reduced odds of SARS-CoV-2 infection. While the effects of antibiotic therapy on the human microbiome is prolonged, the duration of its immunomodulatory effects is unclear. It should be noted that azithromycin has an excellent tissue penetration and it accumulates within tissues and cells, particularly macrophages, with tissue concentrations about 50-fold greater than plasma concentration [29,41]. Its biologic half-life is long, estimated at 35–40 h in humans administered a single dose of 500 mg [29,46].

TMP/SMX (called also cotrimoxazole) was also associated with a significant and independent reduced odd of SARS-CoV-2 infection. In addition to its antimicrobial effects on the human microbiome, TMP/SMX also blocks the stimulation of the formyl peptide receptors, which are expressed on human neutrophils and monocytes, and thus inhibits cytokine production and exerts anti-inflammatory properties [48]. Attempts were therefore made to use TMP/SMX in patients with COVID-19, with reduced severity and mortality in a retrospective analysis [48], case series [49], and in an interim analysis of a controlled study [50]. These effects of TMP/SMX might explain its association with reduced rates of SARS-CoV-2 infection.

Fluoroquinolones were associated with a significantly decreased rate of SARS-CoV-2 infection in the univariate analysis but not in the multivariate model, which included diseases that are related to the risk of the infection. This probably implies that the fluoroquinolones were more often used in patients with underlying medical conditions. Multiple immune modulatory activities of fluroquinolones are well-documented, including a decrease in cytokine release and attenuation of the inflammatory response [31,32,33]. They also have anti-viral activities, including against SARS-CoV-2, by binding to its protease [51]. Because of these properties, fluoroquinolones have been proposed as adjuncts in the treatment of SARS-CoV-2-associated pneumonia [52]. Bronchial asthma was associated with a reduced rate of SARS-CoV-2 infection, as we have reported and discussed previously in a large-scale study that has focused on this condition [53].

### 4.2. Strengths and Limitations

The main strength of our study is its being large-scale, population-based and performed on real-world data, with the ability to collect comprehensive demographic and clinical information to build a stringently matched control group and to perform a multivariate analysis with potential confounders. The advanced digital systems enabled us to determine individual antibiotic consumption prior to the SARS-CoV-2 infection by utilizing cutting-edge tracking methodologies. The big sample size was sufficient to reach statistical significance while controlling for confounders.

The study has several limitations. The major one relates to its observational and retrospective methodology, with the possibility that some unrecognized biases could have affected the results. We therefore used the term association, not causality, to describe our findings. Our analysis of antibiotic consumption was based on the acquisition of the antibiotic agent from the pharmacy, but we could not confirm the actual antibiotic use by the individual, and the duration of use. As our population was largely Caucasian, our findings may not be automatically generalized to other ethnic populations. Because the study is based on a single HMO, although nation-wide, a selection bias is possible.

## 5. Conclusions

This population-based comprehensive study documented a new finding that previous consumption of several antibiotic agents was significantly and independently associated with reduced or increased rates of SARS-CoV-2 infection. Two plausible mechanisms, and the interplay between them, are the prolonged antibiotic effects on the human microbiome and on the immune response. Further studies, preferably prospective, are needed to replicate the finding in other populations, determine the causality, and elucidate the mechanisms involved.

## Figures and Tables

**Table 1:** Demographic characteristics of the cases and control groups *.

Variable	Variable Sub-Group	Documented Infection with SARS-CoV-2 (Cases)	No Documented INFECTION with SARS-CoV-2 (Controls)
**All group, N**		**31,260**	**125,039**
**Gender *n* (%)**	**Male**	15,512 (49.6%)	62,048 (49.6%)
	**Female**	15,748 (50.4%)	62,991 (50.4%)
**Age (years), mean (SD)**		30.17 (20.13)	30.13 (20.19)
**Age category, *n* (%)**	**0–2 years**	1227 (3.9%)	4908 (3.9%)
	**3–9 years**	3544 (11.3%)	14,176 (11.3%)
	**10–18 years**	6178 (19.8%)	24,712 (19.8%)
	**19–29 years**	6611 (21.1%)	26,444 (21.1%)
	**30–39 years**	4184 (13.4%)	16,736 (13.4%)
	**40–49 years**	3460 (11.1%)	13,840 (11.1%)
	**50–59 years**	2894 (9.3%)	11,576 (9.3%)
	**60–69 years**	1915 (6.1%)	7659 (6.1%)
	**70–79 years**	796 (2.5%)	3184 (2.5%)
	**80–89 years**	357 (1.1%)	1428 (1.1%)
	**≥90 years**	94 (0.3%)	376 (0.3%)
**Family status, *n* (%)**	**Married**	9080 (29.0%)	34,475 (27.6%)
	**Single**	1989 (6.4%)	9306 (7.4%)
	**Divorced**	379 (1.2%)	1999 (1.6%)
	**Widower/Widow**	179 (0.6%)	757 (0.6%)
	**Unknown**	19,633 (62.8%)	78,502 (62.8%)
**Ethnic group *n* (%)**	**Arab**	6721 (21.5%)	26,884 (21.5%)
	**Secular Jews**	13,842 (44.3%)	55,368 (44.3%)
	**Ultra-orthodox Jews**	10,697 (34.2%)	42,787 (34.2%)
**Level of socioeconomic status, mean (SD)**		7.19 (3.49)	7.54 (3.63)

* All *p*-values between the groups are not significant (>0.05).

**Table 2:** Clinical variables in the cases and control groups *.

Variable	Variable Sub-Group	Documented Infection with SARS-CoV-2 (Cases)	No Documented Infection with SARS-CoV-2 (Controls)
**Smoking status *n* (%)**	**Non-smoker**	17,569 (87.3%)	69,875 (87.2%)
	**Past smoker**	123 (0.6%)	501 (0.6%)
	**Smoker**	2436 (12.1%)	9747 (12.2%)
	**missing**	11,132 (35.6%)	44,916 (35.9%)
**Weight (Kg), mean (SD)**		61.46 (26.29)	61.11 (26.45)
	**missing**	2175 (6.96%)	8509 (6.81%)
**Height (cm), mean (SD)**		158.03 (21.55)	157.69 (22.11)
	**missing**	3614 (11.56%)	14,524 (11.62%)
**Body mass index (BMI), mean (SD)**		24.22 (6.30)	24.19 (6.29)
	**missing**	3633 (11.62%)	14,604 (11.68%)
**Body mass index (BMI) category, N (%)**	**<18.5 Underweight**	5498 (20.0%)	22,017 (20.0%)
	**18.5–24, normal**	9874 (35.9%)	39,433 (35.9%)
	**25–29, overweight**	7247 (26.3%)	28,906 (26.3%)
	**30–34, obese I**	3524 (12.8%)	14,084 (12.8%)
	**35–40, obese II**	1012 (3.7%)	4051 (3.7%)
	**>40, obese III**	349 (1.3%)	1393 (1.3%)
	**missing**	3756 (12.0%)	15,155 (12.1%)
**Diabetes mellitus, *n* (%)**		1876 (6.0%)	7504 (6.0%)
**Systolic blood pressure (mmHg), mean (SD)**		118.76 (15.61)	118.64 (15.77)
	**missing**	8205 (26.25%)	32,653 (26.11%)
**Diastolic blood pressure (mmHg), mean (SD)**		72.29 (10.07)	72.33 (10.14)
	**missing**	8207 (26.25%)	32,658 (26.12%)
**Pulse (*n*/min), mean**		79.44 (14.01)	79.25 (14.19)
	**missing**	9657 (30.89%)	38,084 (30.46%)
**Blood pressure category**	**Hypertension**	5959 (25.81%)	23,959 (25.89%)
	**Normal blood pressure**	17,132 (74.2%)	68,558 (74.1%)
	**missing**	8169 (26.1%)	32,522 (26.0%)

* All *p*-values between the groups are not significant (>0.05).

**Table 3:** Laboratory characteristics of the cases and control groups *.

Variable	Variable Sub-Group	Documented Infection with SARS-CoV-2 (Cases)	No Documented Infection with SARS-CoV-2 (Controls)
**Serum creatinine (mg/dl), mean (SD)**		0.69 (0.33)	0.69 (0.37)
	**missing**	7462 (23.87%)	28,975 (23.17%)
eGFR * (mL/min), mean (SD)		147.25 (114.08)	148.75 (121.76)
	**missing**	7475 (23.91%)	29,015 (23.20%)
eGFR * category (mL/min), N (%)	**<15**	37 (0.2%)	190 (0.2%)
	**16–29**	51 (0.2%)	193 (0.2%)
	**30–44**	114 (0.5%)	548 (0.6%)
	**45–59**	308 (1.3%)	1398 (1.5%)
	**60–89**	4124 (17.7%)	17,347 (18.5%)
	**(Normal)**	18,655 (80.1%)	73,930 (79.0%)
	**missing**	7971 (25.5%)	31,433 (25.1%)
**Hemoglobin A1C, % mean (SD)**		5.51 (0.88)	5.49 (0.84)
	**missing**	18,133 (58.01%)	72,126 (57.68%)
HDL * (mg/dl), mean (SD)		49.06 (11.89)	49.15 (11.95)
	**missing**	11,097 (35.50%)	43,207 (34.55%)
LDL * (mg/dl), mean (SD)		108.73 (33.36)	109.29 (33.76)
	**missing**	11,133 (35.61%)	43,321 (34.65%)
**Triglycerides (mg/dl), mean (SD)**		109.47 (71.91)	110.66 (72.37)
	**missing**	10,549 (33.75%)	41,036 (32.82%)

* All *p*-values between the groups are not significant (>0.05); eGFR, estimated glomerular filtration rate; HDL, high density lipoprotein cholesterol; LDL, low density lipoprotein cholesterol.

**Table 4:** The association between antibiotic consumption and SARS-C0V-2 infection.

Antimicrobial Agent or Class	Use in Cases N (%)	Use in Controls N (%)	Odds Ratio [95% CI]	*p*-Value
A. The association between antibiotic consumption 30–365 days before SARS-CoV-2 testing and SARS-CoV-2 infection. For convenience, significant associations with decreased rates of SARS-CoV-2 infection are colored in light green and those associated with increased rates are colored in pink.				
**Trimethoprim-sulfamethoxazole**	100 (0.32)	532 (0.46)	0.751 [0.600, 0.932]	0.0082
**Fluoroquinolones, all**	1024 (3.28)	4425 (3.54)	0.923 [0.861, 0.989]	0.0229
**Levofloxacin**	153 (0.49)	724 (0.58)	0.845 [0.704, 1.007]	0.0624
**Ciprofloxacin**	756 (2.42)	3272 (2.62)	0.922 [0.850, 1.000]	0.0482
**Macrolides, all**	2039 (6.52)	8850 (7.08)	0.916 [0.871, 0.963]	0.0005
**Azithromycin**	1297 (4.15)	5919 (4.73)	0.871 [0.819, 0.926]	<0.0001
**Penicillins, all**	7686 (24.59)	30,412 (24.32)	1.014 [0.986, 1.044]	0.3310
**Amoxicillin**	4728 (15.13)	18,729 (14.98)	1.012 [0.977, 1.047]	0.5181
**Penicillin-beta-lactamase Inhibitor**	2994 (9.58)	12,186 (9.75)	0.981 [0.940, 1.023]	0.3755
**Phenoxymethylpenicillin**	1044 (3.34)	3784 (3.03)	1.107 [1.032, 1.187]	0.0046
**First generation cephalosporins**	1245 (3.98)	5008 (4.01)	0.994 [0.932, 1.059]	0.8718
**Second generation cephalosporins**	1306 (4.18)	5502 (4.40)	0.947 [0.890, 1.008]	0.0855
**Third generation cephalosporins**	129 (0.41)	439 (0.35)	1.176 [0.958, 1.435]	0.1147
B. The association between antibiotic consumption 30–180 days before SARS-CoV-2 testing and SARS-CoV-2 infection. For convenience, significant associations with decreased rates of SARS-CoV-2 infection are colored in light green and those associated with increased rates are colored in pink.				
**Trimethoprim-sulfamethoxazole**	42 (0.13)	290 (0.23)	0.579 [0.408, 0.802]	0.0005
**Fluoroquinolones, all**	461 (1.47)	2073(1.65)	0.888 [0.800, 0.983]	0.0212
**Levofloxacin**	38 (0.12)	264 (0.21)	0.575 [0.398, 0.811]	0.0008
**Ciprofloxacin**	365 (1.16)	1547 (1.23)	0.943 [0.839, 1.058]	0.3281
**Macrolides, all**	538 (1.72)	2411 (1.92)	0.891 [0.809, 0.979]	0.0156
**Azithromycin**	276 (0.88)	1291 (1.03)	0.854 [0.747, 0.974]	0.0172
**Penicillins, all**	3245 (10.38)	12,685 (10.14)	1.026 [0.985, 1.069]	0.2175
**Amoxicillin**	1826 (5.84)	7092 (5.67)	1.032 [0.978, 1.088]	0.2466
**Penicillin**-**beta-lactamase Inhibitors**	1294 (4.12)	5245 (4.19)	0.986 [0.926, 1.050]	0.6698
**Phenoxymethylpenicillin**	363 (1.16)	1273 (1.01)	1.142 [1.013, 1.285]	0.0273
**First generation Cephalosporins**	573 (1.83)	2329 (1.86)	0.984 [0.896, 1.079]	0.7430
**Second generation cephalosporins**	503 (1.61)	2114 (1.69)	0.951 [0.860, 1.049]	0.3243
**Third generation Cephalosporins**	49 (0.15)	179 (0.14)	1.095 [0.781, 1.510]	0.5623
C. The association between antibiotic consumption 15–365 days before SARS-CoV-2 testing and SARS-CoV-2 infection. For convenience, significant associations with decreased rates of SARS-CoV-2 infection are colored in light green and those associated with increased rates are colored in pink.				
**Trimethoprim-sulfamethoxazole**	105 (0.36)	551 (0.44)	0.761 [0.612, 0.940]	0.0095
**Fluoroquinolones, all**	1059 (3.38)	4606 (3.68)	0.917 [0.856, 0.982]	0.0122
**Levofloxacin**	157 (0.50)	743 (0.59)	0.844 [0.706,1.005]	0.0545
**Ciprofloxacin**	783 (2.50)	3421 (2.73)	0.913 [0.843, 0.988]	0.0233
**Macrolides, all**	2082 (6.66)	9026 (7.21)	0.917 [0.873, 0.964]	0.0005
**Azithromycin**	1317 (4.21)	5995 (4.79)	0.873 [0.821, 0.929]	<0.0001
**Penicillins, all**	7871 (25.17)	31,126 (24.89)	1.015 [0.987, 1.045]	0.2960
**Amoxicillin**	4853 (15.52)	19,172 (15.33)	1.015 [0.980, 1.050]	0.4000
**Combinations of Penicillins and beta-lactamase Inhibitors**	3080 (9.85)	12,596 (10.07)	0.976 [0.936, 1.017]	0.2469
**Phenoxymethylpenicillin**	1072 (3.42)	3878 (3.10)	1.109 [1.035, 1.189]	0.0034
**First generation Cephalosporins**	1295 (4.14)	5244 (4.19)	0.987 [0.927, 1.051]	0.6930
**Second generation Cephalosporins**	1347 (4.30)	5673 (4.53)	0.947 [0.891, 1.007]	0.0818
**Third generation Cephalosporins**	130 (0.14)	449 (0.35)	1.159 [0.945, 1.412]	0.1449

**Table 5:** Multivariable analysis with adjusted Odds ratio (adj. OR) and 95% confidence interval (95% CI) for the independent association between previous antibiotic consumption and underlying diseases and SARS-CoV-2 infection. Variables significantly different between the groups by univariate analysis were entered into the multivariate model.

Antimicrobial Agent or Underlying Disease	Use in Cases N (%)	Use in Controls N (%)	Adjusted Odds Ratio [95% CI]	*p*-Value
**Trimethoprim-sulfamethoxazole**	100 (0.32)	532 (0.46)	0.783 [0.632, 0.971]	0.0256
**Fluoroquinolones, all**	1024 (3.28)	4425 (3.54)	0.955 [0.891, 1.024]	0.1986
**Azithromycin**	1297 (4.15)	5919 (4.73)	0.882 [0.829, 0.938]	<0.0001
**Phenoxymethylpenicillin**	1044 (3.34)	3784 (3.03)	1.110 [1.036, 1.191]	0.0032
**Attention deficit-hyperactivity disorder**	2811 (8.99)	11,937 (9.55)	0.930 [0.891, 0.971]	0.0011
**Asthma**	1988 (6.36)	8512 (6.81)	0.943 [0.896, 0.992]	0.0240
**Ischemic heart Disease**	694 (2.22)	3191 (2.63)	0.859 (0.787, 0.038)	0.0007
**Congestive heart failure**	225 (0.72)	1063 (0.85)	0.921 (0.791, 1.073)	0.2904
**Chronic obstructive pulmonary disease**	473 (1.51)	2140 (1.71)	0.944 (0.851, 1.047)	0.2745
**Inflammatory bowel disease**	188 (0.60)	892 (0.71)	0.859 (0.733, 1.006)	0.0587
**Dementia**	248 (0.79)	807 (0.65)	1.306 91.146, 1.489)	<0.0001
**Solid tumors**	518 (1.7)	2536 (2.0)	0.831 [0.755, 0.916]	0.0002

## Data Availability

All statistical analyses are available upon requests. Because of ethical and privacy issues, the patients’ data cannot be shared.
